# Modelling the evolution of the archeal tryptophan synthase

**DOI:** 10.1186/1471-2148-7-59

**Published:** 2007-04-10

**Authors:** Rainer Merkl

**Affiliations:** 1 Institut für Biophysik und Physikalische Biochemie, Universität Regensburg, 93040 Regensburg, Germany

## Abstract

**Background:**

Microorganisms and plants are able to produce tryptophan. Enzymes catalysing the last seven steps of tryptophan biosynthesis are encoded in the canonical *trp *operon. Among the *trp *genes are most frequently *trpA *and *trpB*, which code for the alpha and beta subunit of tryptophan synthase. In several prokaryotic genomes, two variants of *trpB *(named *trpB1 *or *trpB2*) occur in different combinations. The evolutionary history of these *trpB *genes is under debate.

**Results:**

In order to study the evolution of *trp *genes, completely sequenced archeal and bacterial genomes containing *trpB *were analysed. Phylogenetic trees indicated that TrpB sequences constitute four distinct groups; their composition is in agreement with the location of respective genes. The first group consisted exclusively of *trpB1 *genes most of which belonged to *trp *operons. Groups two to four contained *trpB2 *genes. The largest group (*trpB2_o*) contained *trpB2 *genes all located outside of operons. Most of these genes originated from species possessing an operon-based *trpB1 *in addition. Groups three and four pertain to *trpB2 *genes of those genomes containing exclusively one or two *trpB2 *genes, but no *trpB1*. One group (*trpB2_i*) consisted of *trpB2 *genes located inside, the other (*trpB2_a*) of *trpB2 *genes located outside the *trp *operon. TrpA and TrpB form a heterodimer and cooperate biochemically. In order to characterise *trpB *variants and stages of TrpA/TrpB cooperation *in silico*, several approaches were combined. Phylogenetic trees were constructed for all *trp *genes; their structure was assessed *via *bootstrapping. Alternative models of *trpB *evolution were evaluated with parsimony arguments. The four groups of *trpB *variants were correlated with archeal speciation. Several stages of TrpA/TrpB cooperation were identified and *trpB *variants were characterised. Most plausibly, *trpB2 *represents the predecessor of the modern *trpB *gene, and *trpB1 *evolved in an ancestral bacterium.

**Conclusion:**

In archeal genomes, several stages of *trpB *evolution, TrpA/TrpB cooperation, and operon formation can be observed. Thus, archeal *trp *genes may serve as a model system for studying the evolution of protein-protein interactions and operon formation.

## Background

The synthesis of tryptophan is a common metabolic capability of microorganisms and higher plants, which is not provided by mammals. The prokaryotic *trp *operon encodes the enzymes catalysing the final and pathway-specific steps from chorismate to L-tryptophan. For more than 40 years, the enterobacterial operon has now been the classical model system for studying the evolutionary relation of genes and enzymes (see [1,2] and references therein) as well as gene regulation. Considering gene regulation, several, conceptually quite different mechanisms have been described for the *trp *operon. Most of them were elucidated in bacterial species (see *e.g*. [3-5], and references therein). However, regulation of *trp *operon expression has also been shown for the archaea *Methanothermobacter thermoautotrophicus *[6,7] and *Thermococcus kodakaraensis *[8]. The reason for an elaborated regulation may be the fact that tryptophan is one of the amino acids, whose biochemical synthesis is very expensive [9]. Besides regulation, other features of tryptophan biosynthesis have been studied extensively. The composition of the operon and several aspects of its evolution have been analysed [10], and for each enzyme, at least one 3D-structure has been determined. Taken together, the *trp *operon is besides the ribosomal protein operons one of the best-characterised gene clusters occurring in microorganisms. Its investigation has provided fundamental insights into many aspects of bacterial genetics and enzymology; see [2].

The canonical *trp *operon encodes seven enzymes responsible for the synthesis of L-tryptophan from chorismate. The first reaction is catalysed by the anthranilate synthase, a glutamine amidotransferase, which is a complex consisting of the larger synthase (TrpE) and a smaller glutaminase (TrpG) subunit. The anthranilate phosphoribosyl transferase (TrpD) provides the glutamine amidotransferase function that allows glutamine to serve as the amino donor in anthranilate formation. The two subsequent enzymes, TrpF and TrpC, catalyse the isomerisation of phosphoribosylanthranilate and the synthesis of indole-3-glycerol phosphate, respectively.

TrpA and TrpB constitute the αββα tryptophan synthase complex which catalyses the final reaction from indole-3-glycerole phosphate + L-serine to L-tryptophan + H_2_O. The α subunit (TrpA) cleaves indoleglycerol-3-phosphate to glyceraldehyde-3-phosphate and indole. The latter is transported through a hydrophobic tunnel to the associated β subunit (TrpB), where it is condensed with L-serine to yield L-tryptophan [11]. A sophisticated mechanism of allostery links the α and β monomers of the synthase; see *e.g*. [12].

Several Trp enzymes represent paradigmatically larger classes of proteins having similar function or protein architecture: TrpG is similar to HisH (an enzyme involved in histidine biosynthesis) and other glutaminases of type I glutamine amidotransferases [13]. TrpF, TrpC and TrpA are all (βα)_8 _barrels possessing similar phosphate binding sites [14]. The basic (βα)_8 _barrel is the most common enzyme fold in the PDB database of known protein structures [15].

For the bacterial *trp *genes, the following order was determined: large anthranilate synthase subunit *(trpE)*, small anthranilate synthase subunit *(trpG)*, anthranilate phosphoribosyl transferase *(trpD)*, indole-3-glycerol phosphate synthase *(trpC)*, phosphoribosyl anthranilate isomerase *(trpF)*, tryptophan synthase β subunit *(trpB) *and tryptophan synthase α subunit *(trpA)*, or abbreviated *trpEGDCFBA *[16]. The gene-fusions *trpGD *and *trpEG *have been observed in several species; moreover, in other genomes, the operon is broken up into several gene clusters. In archeal genomes, order of *trp *genes is highly variable. In *Sulfolobus solfataricus*, an intact operon *trpBADFEGC *is observed. In *Haloferax volcanii*, the *trp *operon is divided into two isolated clusters, *trpCBA *and *trpDFEG*, separated by more than 1200 kb. In the genome of *Natronomonas pharaonis*, there exist three homologs of *trpD *and two homologs of *trpB*, *trpE *and *trpG *each. *Pyrococcus horikoshii *completely lacks the genes for tryptophan synthesis (and for other aromatic amino acids).

The genes *trpB*, *trpA *and *trpE, trpG *are frequently in the same order and in close proximity, *i.e*. they comprise the linkage groups *trpBA *and *trpEG*. In both cases, the gene products constitute a bienzyme complex, whose active centres interact with each other. Because they occur in both bacterial and archeal genomes, these linkage groups have been identified as ancestral [16]. A reconstruction of the tentative ancestral *trp *operon is hampered by the observation that *trp *genes are poor phylogenetic reporters. Different rates of evolution, multiple gene duplications and convergent evolution, as a consequence of specific adaptation to environmental demands, may be the reason for inconsistencies seen in comparisons of phylogenies deduced from *trp *genes or rRNA [16]. Therefore, the evolution of each element of the *trp *operon has to be examined separately.

For evolutionary studies, tryptophan synthase is an especially interesting candidate. This enzyme has been analysed for decades in order to understand the structural basis and functional consequences of protein-protein interactions [17]. The isolated TrpA and TrpB proteins form stable, however poorly active α monomers and ββ homodimers, respectively [18,19]. Their assembly to the native αββα complex induces conformational changes in both subunit types, as shown by X-ray crystallography for the *Pyrococcus furiosus *synthase [18]. The result of this communication between the α and β subunits is a reciprocal activation by one to two orders of magnitude [20]. Conformational changes crucial for the allosteric communication between the active sites of the α– and β-subunits have been analysed in detail for the *Salmonella typhimurium *tryptophan synthase; see *e.g*. [21-24].

The role of the β-subunit is of particular importance for the evolution of Trp synthase. For archaea and bacteria, it is known that two variants of *trpB *genes occur, which can clearly be distinguished by their protein sequences [25]. The major group, harbouring proteins of type TrpB1 includes the enzymes of enterobacteria and *Bacillus subtilis*. The minor group (denoted TrpB2) contains many archeal proteins. Most prokaryotes like *E. coli *possess a single *trpB1 *gene. However, in several bacterial and archeal genomes, a combination of one *trpB1 *and one *trpB2 *gene occurs. In addition, some species exist, which have only one or two *trpB2*, but no *trpB1 *gene. This variety prompted us to characterise the evolution of TrpB and its interaction with TrpA in detail, both biochemically and *in silico*.

Based on biochemical findings, a model for the evolution of the tryptophan synthase complex has recently been introduced [26]. This model assumes the existence of an ancient and non operon-based *trpB2*. After duplication, only one *trpB2 *gene presumably has been integrated into the *trp *operon. Differences in evolutionary pressure may have been responsible for the divergence of non operon- and operon-based *trpB *genes. The coevolution with *trpA *may have led to a better adapted *trpB1*. The data on complex formation and subunit activation led us consider existing *trpB *variants as representatives of evolutionary steps in the postulated model.

In this study, I have assessed this model by phylogenetic methods. Two basic questions have been addressed: *i*) What is the evolutionary relationship of *trpB1 *and *trpB2*? *ii*) How did extant archeal *trp *operons evolve? Extending previous work [25], I will discuss novel hypotheses concerning the properties of TrpB2 and operon formation. Based on the content of 26 completely sequenced archeal genomes, comparative analyses of *trp *sequences, and their locations in genomes will be reported in order to reconstruct the evolution of TrpB-type subunits and of the coevolution of TrpA/TrpB. It will be shown that TrpB2 variants represent different stages of TrpA/TrpB cooperation and that TrpB2 is favoured over TrpB1 in certain environments. Moreover, TrpB2 has features of a more ancient TrpB variant.

## Results and Discussion

### Assessing the composition of *trp *gene clusters

In order to describe the composition of *trp *regulons in a quantitative manner and to compare their content in archeal and bacterial genomes, AMIGOS [27] was used. By comparing genomes, this program identifies gene clusters and rates each individual cluster element with a *cons*_*CL*_-score. The *cons*_*CL*_-score of an individual gene depends on *i*) the occurrence of this gene in a given gene cluster and *ii*) the global similarity of the genomes harbouring these clusters. Thus, individual scores assess both the relatedness of genomes and the frequency with which individual genes are members of a cluster. The higher a score, the more pronounced is the occurrence of an individual gene in a given gene cluster. Table 1 lists *cons*_*CL*_-scores for elements of archeal and bacterial *trp *operons. The numbers indicate that in bacteria the clustering of *trpA *and *trpB1 *was stronger than that of all other *trp *genes. In archeal genomes, the clustering of *trpE *and *trpG *was most prominent. A reason for the lower score of *trpB *in archeal *trp *operons was the occurrence of two *trpB *variants (*trpB1 *and *trpB2*) in these species. The scores signalled that *trpB1 *was more frequently part of an *trp *operon than *trpB2*. Moreover, the score for *trpA *was lower than that of *trpE *or *trpG*. It follows for archaea that *trpA *and *trpB *are less strictly integrated into *trp *operons than in bacteria. This suggests that either evolutionary pressure responsible for operon formation is less pronounced or that additional selective forces disfavour the integration of *trpA *and *trpB *into certain archeal *trp *operons.

**Table 1:** *cons*_*cl *_scores for *trp *genes

***cons*_*Cl *_– values**		**Protein**	**COG #**	**Function**
Archaea	Bacteria			

2.0	2.8	TrpE	COG0147	anthranilate/para-aminobenzoate synthases comp. I
2.1	2.7	TrpG	COG0512	anthranilate/para-aminobenzoate synthases comp. II
1.8	2.3	TrpF	COG0135	phosphoribosylanthranilate isomerase
1.8	2.9	TrpC	COG0134	indole-3-glycerol phosphate synthase
1.9	3.0	TrpA	COG0159	tryptophan synthase alpha chain
1.4	3.0	TrpB1	COG0133	tryptophan synthase beta chain
0.6	-	TrpB2	COG1350	paralogue of TrpB
1.9	2.6	TrpD	COG0547	anthranilate phosphoribosyltransferase

Note: The first two columns list score values deduced from representative sets of archeal and bacterial genomes. Columns three and four list protein names, COG numbers, and protein function. The *cons*_*cl*_-scores were determined by using AMIGOS [27]. COG numbers indicate orthologous gene clusters as defined in the COG database [29].

It has been hypothesised that TrpB2 possesses a second function and acts as a serine deaminase [25]. This prediction has been deduced from the analysis of phyletic patterns, *i.e*. the absence of an encoded serine deaminase function in certain genomes. However, it has been shown that TrpB1 of *Thermotoga maritima *and TrpB2_o proteins of *Sulfolobus solfataricus *and *T. maritima *have poor serine deaminase activities [26]. An alternative method of non-homologous gene annotation is the exploitation of gene neighbourhoods [28], as *e.g*. implemented with AMIGOS. For *trpB2*, AMIGOS did not detect a second conserved gene neighbourhood besides the one constituting *trp *operons. Thus, no clues for an additional function besides tryptophan synthesis have been deduced for *trpB2 *by this approach.

### A naming code for *trpB *genes

The two variants of *trpB *occur in various genomes in different combinations [25]. In order to facilitate the analysis of phylogenetic trees, a naming scheme was introduced. Names of genes and gene products were generated according to the scheme *SPECIES_LOC|TYPE|TAX*. Here, *SPECIES *is an abbreviation of the species name (see Materials). *LOC *indicates the position of the specific *trpB *gene relative to a putative *trp *operon (more precisely: relative to a *trpA *gene). If two *trpB *genes occur in a genome, they were labelled _i (if the gene was located inside the *trp *operon) or _o (if located outside the operon). If only a single *trpB *gene occurred in the genome, it was labelled _s, if the gene was linked to *trpA*, and it was labelled _S, if it was separate from *trpA*. *TYPE *indicates the gene type. It is 1 for *trpB1 *and 2 for *trpB2*. Finally, *TAX *gives the taxonomical classification. It is C for *Crenarchaeota*, E for *Euryarchaeota *and B for *Bacteria*. The following examples explain how to resolve sequence names: Aperni_o2C was used to name a *trpB *gene in the genome of *Aeropyrum pernix *(Aperni), which occurred outside the *trp *operon (_o) and was of type *trpB2 *(2). As *A. pernix *is a *Crenarchaeota*, the name ends with a C. The _o notation indicates that a second *trpB *gene exists in *A. pernix*. This gene was consequently named Aperni_i2C, as it is a *trpB2 *gene inside the *trp *operon. Note that also pairs like Tmarit_i1B and Tmarit_o2B exist indicating the occurrence of a *trpB1 *gene inside and a *trpB2 *gene outside the *trp *operon. Sacido_s2C is the designation of a *trpB2 *gene located inside the *trp *operon. As *Sulfolobus acidocaldarius *possesses only one *trpB *gene, it was labelled with a _s. Since *Thermoplasma volcanium *possesses only one *trpB *gene, which is non operon-based and of type *trpB2*, this gene was named Tvolc_S2E. Designations of the encoded proteins were assigned in a corresponding way.

### Determining the occurrence of *trpB *genes

In order to determine the distribution of *trpB *variants, the COG [29] and the STRING database [30] were used. For all completely sequenced archeal and bacterial genomes, their occurrence was determined and their location was identified. Depending on the occurrence of *trpB *variants, archeal species were grouped into five categories, named species-types in the following. Note that these species-types characterise the content of genomes. Links to the above naming scheme for genes are gene location and type.

As Table 2 shows, there were six archeal genomes possessing a single *trpB *gene of class *trpB1 *(*s1 *or *S1 *species), four genomes with a single *trpB *gene of class *trpB2 *(*s2 *or *S2 *species), five genomes harbouring one operon-based and one additional, non operon-based *trpB2 *each (*i2_o2 *species), ten species of type *i1_o2 *(one operon-based *trpB1 *and one additional *trpB2 *gene) and one species possessing one operon-based and at least one non operon-based *trpB1 *gene (*i1_o1 *species). The most frequent combination (10 out of 26) was an operon-based *trpB1 *and a non operon-based *trpB2 *gene (*i1_o2 *species). *N. pharaonis *was the only archeal species of type *i1_o1*. All five completely sequenced *Crenarchaeota *possess exclusively genes of class *trpB2*.

**Table 2:** Classifying known archeal genomes according to the occurrence of *trpB *genes

***S2 *(3), *s2 *(1)**	***i2_o2 *(5)**	***i1_o2 *(10)**	***i1_o1 *(1)**	***S1 *(1), *s1 *(5)**
*S. acidocaldarius*, s2C 3, TA *T. volcanium*, S2E 3, TA *T. acidophilum*, S2E 3, TA *P. horikoshii*, S2E 2, HT	*A. pernix*, i2C 4, o2C 2, HT *P. aerophilum*, i2C 3, o2C 4, HT *P. torridus*, i2E 2, o2E 2, TA *S. solfataricus*, i2C 3, o2C 3, TA *S. tokodaii*, i2C 3, o2C 4, TA	*A. fulgidus*, i1E 2, o2E 6, HT *M. acetivorans*, i1E 1, o2E 5, MS *M. barkeri*, i1E 1, o2E 4, MS *M. burtonii*, i1E 1, o2E 4, MS *M. hungatei*, i1E 1, o2E 4, MS *M. mazei*, i1E 1, o2E 5, MS *M. thermoautotrophicus*, i1E 1, o2E 3, TP *P. abyssi*, i1E 3, o2E 2, HT *P. furiosus*, i1E 3, o2E 2, HT *T. kodakaraensis*, i1E 2, o2E 2, HT	*N. pharaonis*, i1E 1, o1E 0, HP	*M. kandleri*, S1E 4, YP *Halobacterium*, s1E 1, HP *H. marismortui*, s1E 1, HP *M. maripaludis*, s1E 1, MS *M. stadtmanae*, s1E 1, MS *M. jannaschii*, s1E 2, YP
2.75	*i2: *3.0, *o2: *3.0	*i1: *1.6, *o2: *3.7	*i1: *1.0, *o1: *0.0	1.6

Note: The occurrence and the location of *trpB *genes were coded according to the following scheme used in the top line: *S2 *species possess exactly one, non operon-based *trpB2 *gene, *s2*: ditto, the gene is located inside the *trp *operon. *trpB1 *was treated analogously. *i2_o2 *are species possessing an *trpB2 *gene inside and a second *trpB2 *outside the operon, *i1_o2 *are species with an operon-based *trpB1 *and a non operon-based *trpB2*, and *i1_o1 *are species possessing an operon-based and at least one non operon-based *trpB1*. The number of genomes having the same species-type is given in brackets. The acronyms following the species name classify *trpB *genes; see Results. Numbers give tryptophan codons occurring in the respective gene. The abbreviations indicate hyperthermophilic (HT), thermoacidophilic (TA), thermophilic (TP), mesophilic (MS), halophilic (HP), or hyperthermophilic + halophilic species (YP). The last line of the table gives mean values for the occurrence of tryptophan codons.

Bacterial species did not contribute species-types noticeably different from those observed among archaea (data not shown). Both *Geobacter *species represent special cases most plausibly explained by ongoing genomic rearrangements: Gsulfu_i2B is an operon-based *trpB2 *gene of type TrpB2_o. The *trp *operon of *G. sulfurreducens *harbours both a *trpB1 *and a *trpB2 *gene. According to the annotation, the *trpB1 *gene (Locus tag GSU2375) contains a frameshift and is annotated as a pseudogene [31]. A direct neighbour of *trpB1 *in *G. metallireducens *is a transposase, making a recent transfer of this gene plausible. In comparison to archaea, the occurrence of *trpB2 *was less frequent in bacterial genomes and none contained exclusively *trpB2 *genes.

### Assessing phylogenetic relationship of *trp *genes

**Figure 1:**
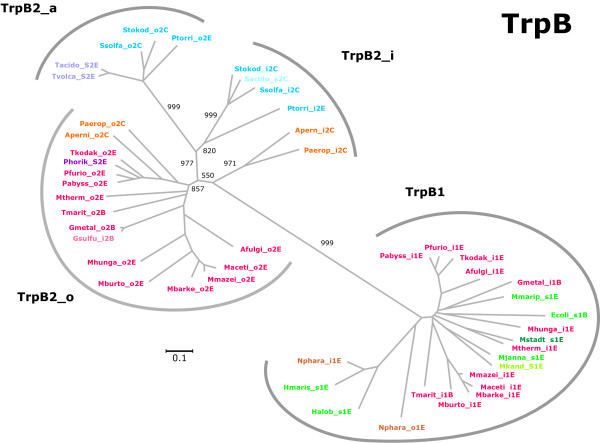
**Phylogenetic tree of TrpB sequences**. Using archeal and bacterial TrpB sequences, a multiple sequence alignment was generated and an unrooted phylogenetic tree was constructed. Proteins were labelled according to the naming scheme introduced in the Results section. Subtrees were marked according to the sequence type (TrpB1 or TrpB2). TrpB2 sequences span three subtrees; clustering is in agreement with the location of genes. TrpB2_o proteins are all encoded outside operons; 14 out of 16 originate from species that possess an operon-based *trpB1 *in addition. TrpB2_i proteins are encoded inside operons. Each of these genes is accompanied by a non operon-based *trpB2*. TrpB2_a sequences occur exclusively in genomes that have a single *trpB2 *gene or occur as a second *trpB2 *outside an operon in combination with a *trpB2_i *gene. The numbers are bootstrap values resulting from 1000 replications. Gene names are colour-coded. Blue colours indicate genes occurring in *S2 *(violet), *s2 *(light blue) and those *i2_o2 *species, which possess *trpB2_a *or *trpB2_i *genes (dark blue). Orange colours designate *trpB2_i *and *trpB2_o *genes. Red colours signify genes of *i1_o2*, *S2*, or *s2 *species, and green colours mark genes of *s1 *(light green) or *S1 *(dark green) species. The names of the two *trpB1 *copies occurring in *N. pharaonis *are printed in brown. For acronyms of species-types, see legend of Table 2. The length of the horizontal bar corresponds to 0.1 substitutions per site.

**Figure 2:**
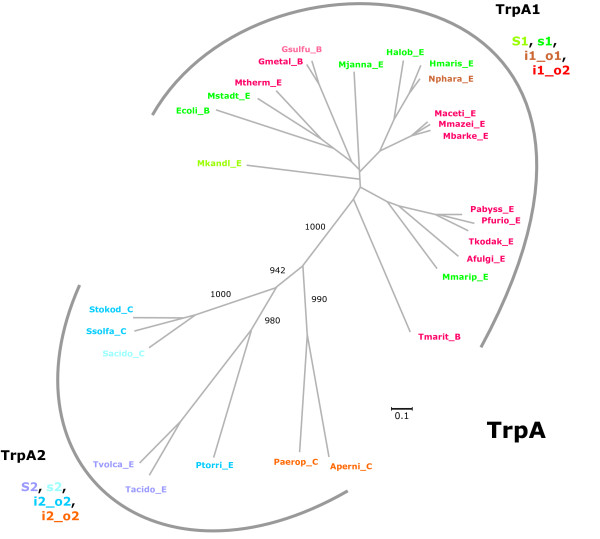
**Phylogenetic tree of archeal and bacterial TrpA sequences**. The two subtrees cluster genomes, which encode at least one *trpB1 *gene (*S1*, *s1, i1_o1*, or *i1_o2 *species) or which possess only genes of type *trpB2 *(*S2, s2*, *i2_o2 *species). The clusters were named TrpA1 or TrpA2, respectively. For abbreviations of sequence names, see Results. For colour code, see legend of Figure 1. For the acronyms of species-types, see legend of Table 2.

**Figure 3:**
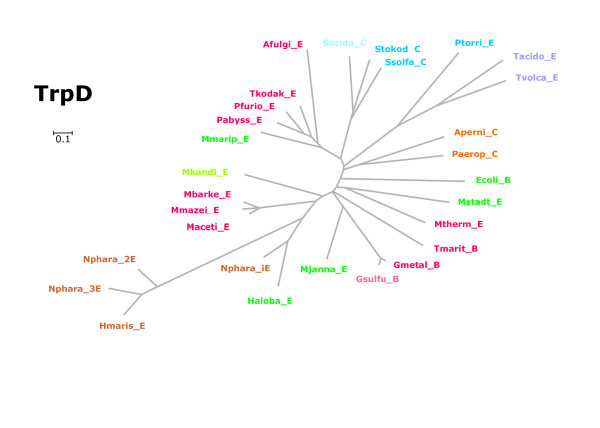
**Phylogenetic tree of TrpD sequences**. Archeal and bacterial protein sequences were used to construct the unrooted tree. The last letter of the acronyms indicates the taxonomical position of the species. "E" marks *Euryarchaeota*, "C" *Crenarchaeota*, and "B" bacterial species. The three TrpD sequences of *N. pharaonis *are designated as _1E, _2E, and _3E. For colour code and abbreviations, see legend of Figure 1.

**Figure 4:**
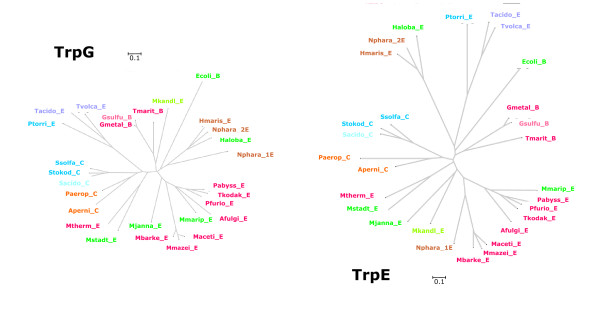
**Phylogenetic trees for TrpG and TrpE sequences**. Archeal and bacterial protein sequences were used to construct the unrooted tree. The last letter of the acronyms indicates the taxonomical position of the species. "E" marks *Euryarchaeota*, "C" *Crenarchaeota*, and "B" bacterial species. The two TrpE sequences of *N. pharaonis *are designated as _1E, _2E. For colour code and abbreviations, see legend of Figure 1.

Sequences originating from all archaea and several representative bacteria were selected for a phylogenetic classification of *trp *genes. Multiple sequence alignments were created by using M-Coffee [32], and trees were constructed and evaluated using SplitsTrees [33]. Figures 1, 2, 3, 4 are plots of unrooted trees generated for protein sequences of TrpA, TrpB, TrpD, TrpE, and TrpG. In order to assess the statistical strength of individual edges, bootstrap resampling was used. For relevant edges, bootstrap values were plotted; see Figures 1, 2, 3, 4. The trees were analysed in detail, as follows.

#### TrpB

In agreement with previous findings [25], TrpB1 and TrpB2 clearly fall into two distinct groups. This distinction was supported by a high bootstrap value; see Figure 1. Moreover, among TrpB2 sequences a finer sub-clustering could be deduced, which was in agreement with the location of the genes. One group (labelled TrpB2_o) consisted of products of *trpB2 *genes not located in operons. 14 out of 16 elements were TrpB2 sequences originating from *i1_o2 *species, *i.e*. species possessing besides an isolated lying *trpB2 *an additional, operon-based *trpB1*. The genes Paerip_o2C and Aperni_o2C of the two *i2_o2 *species *Pyrobaculum aerophilum *and *A. pernix *belonged to this group too. These two species possess a *trp *operon containing a *trpB2 *gene. Bacterial TrpB2_o sequences, which originated from the *i1_o2 *species *T. maritima *and *G. metallireducens *did not form an isolated subtree. This finding argues for a common origin of bacterial and archeal *trpB2_o *genes.

The other two subgroups of TrpB2 variants were clearly distinct from the TrpB2_o cluster. The sequences of these clusters originated from archeal *S2 *(*Thermoplasmataceae*), *s2 *or *i2_o2 *species (*Sulfolobaceae, Picrophilus torridus, A. pernix, P. aerophilum*), *i.e*. species possessing exclusively one or two *trpB2 *genes. These sequences formed two clearly separated sets. The first set, named TrpB2_i, subsumes operon-based *trpB2 *genes, and harboured Stokod_i2C, Sacido_s2C, Ssolfa_i2C, Ptorri_i2E, Apern_i2C, and Paerop_i2C. The second set, named TrpB2_a, consisted of Ptorri_o2E, Stokod_o2C, Ssolfo_o2C, Tacido_S2E, and Tvolca_S2E, and subsumed *trpB2 *genes located outside *trp *operons. For *Thermoplasma volcanium *and *Thermoplasma acidophilum*, these *trpB2 *genes were the only *trpB *genes, for *S. solfataricus, S. tokodaii *and *P. torridus*, a second, however distinguishable *trpB2 *gene of type *trpB2_i *was part of the *trp *operon. Proteins of type TrpB2_i formed two finer subgroups: Those of *P. torridus *and the *Sulfolobaceae *resembled more sequences of TrpB2_a. Those of *A. pernix *and *P. aerophilum*, which possess a non operon-based *trpB2_o *gene, were different both from TrpB2_a and from TrpB2_o sequences; see Figure 1. All relevant edges separating these groups are due to their high bootstrap value statistically highly significant.

As a single exception, the genome of *P. horikoshii *did not follow the general classification scheme. It possesses a single *trpB2 *gene, which is of type *trpB2_o *and not – as expected – of type *trpB2_a*. However, this genome lacks all the other *trp *genes, which has been previously interpreted as reductive evolution [10]. The occurrence of a *trpB2_o *gene might be due to the loss of the complete *trp *operon after speciation of *trpB2_i *and *trpB2_o*. The fact that the *P. horikoshii trpB2_o *gene was not affected by the reduction has been considered as an argument for assigning to it an other selective function [25], which has not been identified yet. As noted above, the two bacterial *Geobacter *species represent special cases associated with the presumptive rearrangement of *trp *genes. Briefly, the *trpB *variants can be characterised as follows: *trpB1 *genes occur exclusively in *trp *operons. *trpB2_o *variants represent genes occurring outside operons in those species that have an operon-based *trpB1*. Several archeal species possess exclusively *trpB2 *genes: If only one *trpB2 *gene exists, it is of type *trpB2_a*, if two *trpB2 *genes occur, one is an operon-based *trpB2_i*, the second a *trpB2_a*, or a *trpB2_o *gene.

#### TrpA

Correlated with TrpB speciation, TrpA proteins showed a division into two, statistically highly significant subgroups; see Figure 2. The larger TrpA1 group consisted of TrpA sequences originating from genomes that possess a *trpB1 *gene. Most likely, TrpA1 proteins interact with the operon encoded TrpB1 and thus fall into the same class. The smaller TrpA2 group contained exclusively TrpA proteins of species-types *S2*, *s2*, or *i2_o2*, *i.e*. TrpA proteins whose putative interaction partner is exclusively a TrpB2 protein. The high bootstrap value of 1000 (≜100%) for the central edge emphasises the distinction made between TrpA1 and TrpA2. *S2*, *s2, i2_o2 *species formed three statistically significant subtrees; compare Figure 2. These harboured the TrpA sequences of *(i) Sulfolobaceae*, (*ii*) *Thermoplasmatales *(*T. acidophilum*, *T. volcanium*, *P. torridus*) and (*iii*) *P. aerophilum*, and *A. pernix*. The composition of these groups is in agreement with the TrpB2_a and TrpB2_i groups in Figure 1 and indicates the coevolution of *trpB2 *variants with *trpA*.

#### TrpD, TrpE, and TrpG

In all three trees (see Figures 3 and 4), both the proteins of *Thermoplasmatales *and of the three *Sulfolobaceae *constituted sub-clusters. The edges determined for TrpD or TrpE entries of these species have similar lengths as those calculated for TrpA or TrpB. Especially for the *trpA *and *trpB *genes of these species, an increased rate of evolution has been previously postulated [25]. However, the comparison of trees and edge lengths showed that in these species evolutionary divergence is similarly high for several proteins encoded by the *trp *operon. These findings argue against a specifically increased rate of *trpA *and *trpB *evolution. In general, smaller genomes evolve faster [34]. Therefore, a higher evolutionary rate in the *trp *genes of *Thermoplasmatales *is more plausible explained by a general trend, which is due to their smaller genome size.

Interestingly, no sub-clustering into smaller, distinctly separated groups was observed in TrpE and TrpG, which form like TrpA and TrpB a heteromeric complex. The above finding distinguishes the subunits of tryptophan synthase from those of anthranilate synthase. TrpG was characterised as the evolutionary most stable *trp *protein by the compactness of its phylogenetic tree; see Figure 4.

The three *Euryarchaeota Halobacterium *(*s1*), *Haloarcula marismortui *(*s1*) and *Natronomonas pharaonis *(*i1_s1 *species) constituted an isolated group in all five trees (Figures 1, 2, 3, 4); edge lengths were comparable to those of *s2 *or *i2_o2 *species. This congruence indicates an elevated evolutionary rate for all elements of these *trp *operons. Note that these operons harbour *trpB1 *genes.

### Analysing typical differences in TrpA and TrpB sequences

The phylogenetic tree depicted in Figure 1 illustrates that all TrpB variants can be sorted into four, clearly separated groups. The tree did however not allow to deduce the degree of sequence similarity and to infer whether these differences were subtle sequence variations broadly distributed in the whole sequence or larger indels (inserts or deletions). Table 3 lists the results of pairwise sequence comparisons generated by using BLAST [35]. The selected sequences represent the species-types *S2, i2_o2, i1_o2, s1*, and *S1*. As expected, sequence similarity values are in agreement with tree composition. Importantly, for all pairwise comparisons, more than 25% identical residues were determined. Therefore, all TrpB variants should most probably have the same overall 3D-structure [36].

**Table 3:** Pairwise sequence similarity values of TrpB proteins

	**Ssolfa_i2C**	**Ssolfa_o2C**	**Paerop_i2C**	**Paerop_o2C**	**Afulgi_i1E**	**Afulgi_o2E**	**Tmarit_i1B**	**Tmarit_o2B**	**Mmarip_s1E**	**Mkand_S1E**	**Ecoli_s1B**
**Tacido_S2E**	49, 72, 2	76, 88, 0	47, 65, 2	46, 67, 1	26, 44, 17	46, 64, 2	32, 43, 18	47, 66, 2	28, 43, 12	30, 46, 11	26, 42, 6
**Ssolfa_i2C**	-	54, 75, 2	56, 71, 3	53, 72, 1	34, 48, 14	57, 72, 1	34, 47, 12	54, 73, 1	30, 46, 15	30, 45, 12	28, 42, 14
**Ssolfa_o2C**		-	50, 66, 2	52, 71, 1	33, 50, 13	47, 68, 1	32, 46, 13	48, 70, 1	28, 43, 16	30, 45, 11	27, 40, 10
**Paerop_i2C**			-	57, 68, 3	35, 49, 14	54, 63, 3	35, 50, 12	54, 65, 3	32, 48, 12	34, 46, 15	31, 43, 13
**Paerop_o2C**				-	30, 45, 13	63, 78, 1	32, 46, 13	60, 75, 1	28, 44, 16	33, 46, 11	28, 41, 16
**Afulgi_i1E**					-	31, 44, 11	65, 78, 0	36, 48, 10	65, 85, 0	65, 78, 0	59, 76, 1
**Afulgi_o2E**						-	34, 47, 12	64, 76, 0	30, 45, 9	32, 45, 9	29, 40, 12
**Tmarit_i1B**							-	35, 47, 11	61, 79, 1	64, 79, 1	58, 75, 1
**Tmarit_o2B**								-	34, 46, 9	32, 46, 8	29, 40, 12
**Mmarip_s1E**									-	59, 77, 0	58, 77, 0
**Mkand_S1E**										-	57, 72, 0

Note: The sequences represent species-types *S2, i2_o2, i1_o2, s1*, and *S1*. All pairs were compared by using BLAST with default parameters. For each pair, the fraction of identical residues, similar residues, and inserted gaps is given in percent. For the generation of protein names, see Results.

**Figure 5:**
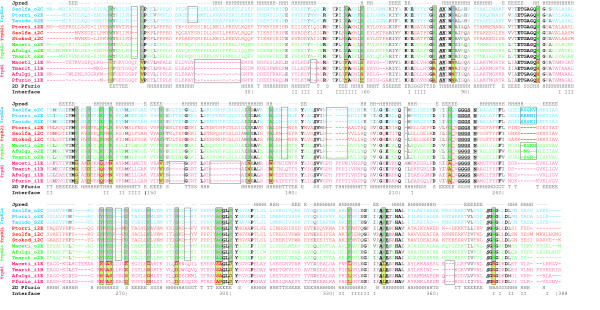
**Multiple sequence alignment of TrpB sequences**. Representatives of the four groups of TrpB sequences were aligned. Ssolfa_o2C, Ptorri_o2E, and Tacido_S2E represent TrpB2_a sequences. Ptorri_i2E, Ssolfa_i2C, and Stokod_i2C represent TrpB2_i, Maceti_o2E, Afulgi_o2E, and Tmarit_o2B represent TrpB2_o sequences. Maceti_i1E, Tmarit_i1B, Afulgi_i1E, and Pfurio_i1E represent TrpB1 sequences. The 2D-structural elements of Pfurio_i1E, as deduced from the PDB file 1WDW, are shown below the sequences, and residues involved in protein interaction with TrpA (I) are assigned under 'Interface'. The line Jpred (top) lists a 2D-prediction of Ssolfa_o2C generated by using the Jpred server [37]. Residues in bold face printing are conserved; black residues are strictly, grey residues are less strictly conserved. Active site residues are plotted in italics; residues in contact with ligands are underlined. These data were deduced from the PDBsum pages [56] and the PISA server [58] of the EMBL-EBI. Residues printed in boxes were predicted by SDPpred [38] as being specific for TrpB1 or TrpB2. See legend of Figure 1 for an explanation of sequence acronyms.

In order to characterise sequence differences in detail, multiple sequence alignments (MSAs) were generated on the basis of a representative selection of TrpA and TrpB sequences. Figure 5 lists for TrpB the MSA, residue conservation, secondary structure and the location of the interface area. Residues interacting with ligands and residues, which are characteristic for TrpB1 and TrpB2 respectively, were labelled. 3D-data were deduced from the X-ray structure of Pfurio_i1E, *i.e*. the operon-based TrpB1 protein of *P. furiosus *[18], which has PDB code 1WDW. For Ssolfa_o2C, the 2D-structure was predicted by using Jpred [37]. SDPpred [38] was employed to identify those residues, which separated TrpB1 and TrpB2 due to their skewed or bimodal distribution. In the following, positions and residues are referenced according to the sequence Pfurio_i1E. Annotations referring active site residues and the interface originate from the PDBsum page and the Macromolecular Structure Database of the EMBL-EBI.

The MSA shows that nearly all differences between TrpB1 and TrpB2 are due to larger indels, in agreement with [25]. Interestingly, an insertion of 2 to 6 residues between positions 243 and 244 occurred coincidently in TrpB2_a and TrpB2_o sequences, *i.e*. exclusively in non operon-based proteins. All considered TrpB1 and TrpB2_i sequences lack this subsequence, which was not predicted as a well-defined 2D-element by Jpred. Several representatives belonging to these two sets of operon-based proteins were shown to interact with TrpA [26,39]. Therefore, it is probable that this putative loop influences the allosteric communication with TrpA. Most residues, which are in contact with ligands in the known TrpB1 structure, were strictly conserved among all TrpB1 and TrpB2 sequences. The only exception is residue C225, which is V225 in TrpB2_a sequences. The active site residues H81, K82, and S371 were strictly conserved, whereas active site residue K162 was conserved only in TrpB1 proteins and active site residue D300 (TrpB1) was an arginine in TrpB2. Several residues of the interface regions, adjacent to active sites and near sites interacting with ligands had a bimodal occurrence pattern distinguishing TrpB1 and TrpB2. Among these were residues 2 and 110, which were strictly conserved tryptophan residues in all TrpB2 proteins. Given its position near the gene start, W2 may assume a function in translation control. W110 succeeds a cluster of strictly conserved residues suggesting a role in stability or protein function.

**Figure 6:**
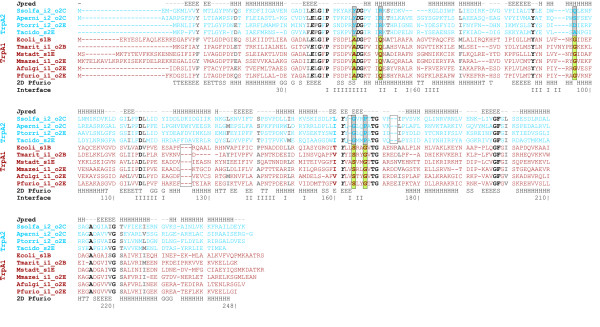
**Multiple sequence alignment of TrpA sequences**. Ssolfa_i2_o2C, Aperni_i2_o2C, and Ptorri_i2_o2C represent TrpA sequences from *i2_o2 *species possessing both an operon-based and a non operon-based *trpB2*. Tacido_s2E represents a species having exclusively an operon-based *trpB2*. Ecoli_s1B and Tmarit_i1_o2B represent bacterial TrpA proteins. Mstadt_s1E is from a species possessing exclusively an operon-based *trpB1 *gene, Mmazei_i1_o2E, Afulgi_i1_o2E and Pfurio_i1_o2E are TrpA sequences from *i1_o2 *species possessing an operon-based *trpB1 *and a non operon-based *trpB2 *gene. Presumably, these TrpA1 proteins interact with a protein of type TrpB1. Below the alignment, the 2D-structure of TrpA of *P. furiosus *(Pfurio_i1_o2E), and residues involved in protein interaction with its TrpB1 (I) are given. The line named Jpred lists a 2D-prediction of Ssolfa_i2_o2C generated by using the Jpred server [37]. Residues printed in bold are conserved; black residues are strictly, grey residues are less strictly conserved. Active site residues are plotted in italics. These data were deduced from the PDBsum pages [56] and the PISA server [58] of the EBI. Residues printed in boxes were predicted by SDPpred [38] as being specific for the two TrpA species.

Figure 6 lists the MSA generated for TrpA sequences. It shows that the active site residues E36, D47, and Y161 are strictly conserved in the TrpA sequences studied. Most evident was a three-residue insertion into TrpA2 sequences following position 125 (numbering deduced from TrpA of *P. furiosus*) as well as deletions at position 162 and between positions 172 and 174. Moreover, most positions showing a bimodal or skewed distribution specific for a *trpA *variant were located near interface regions. In summary, the deviations characterising the two TrpA variants were not as pronounced as those observed in TrpB sequences, however three indels distinguished TrpA1 from TrpA2.

### Frequency of Trp codons in *trpB *genes

It has been postulated that the avoidance of tryptophan residues in enzymes for tryptophan synthesis provides a selective advantage [7] as has been shown for a number of amino acid biosynthetic enzymes [40]. This criterion was also applied to the *trpB *genes by assessing the frequency of tryptophan codons (Table 2). *trpB1 *genes contained one or two tryptophan codons with a mean value of 1.6 both for *S1*, *s1*, and *i1_o2 *species. *trpB2 *genes contained two tryptophan codons or more with a mean of 2.75 for *S2 *species, and 3.0 for *i2_o2 *species. Most pronounced was the difference for *i1_o2 *species. Here, *trpB2 *genes had a mean of 3.7, whereas *trpB1 *genes had a mean of 1.6 tryptophan codons. These *trpB1 *genes showed a habitat-specific imbalance of tryptophan codon occurrence with one in mesophilic species and at least two tryptophan codons in hyperthermophiles. In summary and according to the notion of tryptophan codon avoidance, *trpB2 *genes are less optimised than *trpB1 *genes.

### The composition of archeal *trp *gene clusters

**Figure 7:**
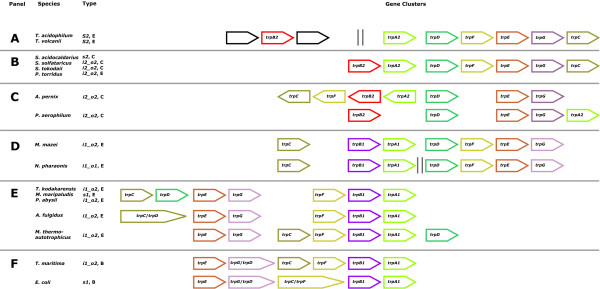
**Gene organisation of archeal and bacterial *trp *gene clusters**. Each panel A – F represents the occurrence and orientation of *trp *genes in the genomes of organisms listed in the second column. The third column gives the species-type and the taxonomical lineage. "E" marks *Euryarchaeota*, "C" *Crenarchaeota*, and "B" bacteria. A vertical double line (Panels A and D) indicates borders of gene clusters separately located in the genome. Open arrows represent hypothetical genes. The arrows are not to scale; gaps of arbitrary length were inserted between genes to allow the alignment of arrows. For acronyms of species-types, see legend of Table 2.

The evolution of individual genes and operon formation proceed in parallel. For the combined analysis of both processes, gene orders of relevant archeal and some bacterial *trp *operons were determined and plotted in Figure 7. In most operons, the gene orders *trpBA *and *trpEG*, respectively, were conserved; however, the arrangement of the linkage groups varied. Figure 7 is organised as six panels A – F. Panel A depicts the *trp *clusters of *Thermoplasmataceae*, which are of type *trpA2DFEGC*; *trpB2 *lies isolated. In *Sulfolobaceae *and *P. torridus *(Panel B), *trpB2 *is the first gene of the gene cluster *trpB2ADFEGC*, which matches the above *trpA2DFEGC *in all positions following *trpB2*. Panel C gives the gene clusters of *A. pernix *and *P. aerophilum*, which possess a *trpB2_i *and a *trpB2_o *gene. In the genome of *A. pernix*, two linkage groups *trpA2B2FC *and *trpDEG *occur; *P. aerophilum *possesses the cluster *trpB2DEGA2*. Panel D lists archeal genomes containing linkage groups *trpCB1A1 *and *trpDFEG*. In *Methanosarcina mazei*, these genes form a single cluster, resulting in *trpCB1A1DFEG*. In *N. pharaonis*, these groups are separated by more than 69kb. In panel E, operons are listed where *trpB1 *lies close to the 3'-terminal end. For *Thermococcus kodakaraensis, Methanococcus maripaludis*, *Archaeoglobus fulgidus *and *Pyrococcus abyssi*, gene order is *trpCDEGFB1A1*. The gene orders in Panel E resemble bacterial operons; two representative examples are plotted in panel F.

It has been argued that simple *trp *clusters may have been unstable until the complexity of regulation and the foundation of a metabolic theme had reached a certain level [10]. Gene clusters observed in *s2 *and *i2_o2 *species can be considered the less evolved stages of cluster organisation; compare Panels A – C. Moreover, the only archeal *trp *gene regulatory systems identified so far are part of the *trp *operons of *M. thermoautotrophicus *[7] and *T. kodakaraensis *[8], which both have a bacterial-like composition.

Besides *Nanoarchaeum equitans, Thermoplasmata *(*T. volcanium*, *T. acidophilum*, and *P. torridus*) possess the smallest archeal genomes sequenced so far. Most plausibly, strong selective pressure associated with the colonised habitat enforces the minimisation of genome size. However, both *Thermoplasma *species possess the gene cluster *trpA2DFEGC*. Therefore, the need for tryptophan synthesis can be taken for granted. The separation of *trpB2 *from the remaining *trp *genes is consistent with a demand for individual gene regulation and expression presumably due to an additional function of TrpB2. Most plausibly, under these constraints, *trpB2 *is the more optimal variant, which is in a specific environment favoured over *trpB1*.

### What is the origin of *trpB *genes?

Recently, TrpA, Tmari_i1B and Tmari_o2B of *T. maritima *have been produced in *E. coli*, purified, and characterised [39]. It has been shown that recombinant TrpA forms an α-monomer, and that both recombinant TrpB proteins form β_2_-homodimers. However, only the operon-encoded Tmari_i1B – but not Tmari_o2B – associated with TrpA to constitute the conventional αββα tryptophan synthase complex in which both subunits reciprocally activate each other. An analogous experiment has been carried out for genes of *S. solfataricus *[26]. The results have shown that Ssolfa_i2C – but not Ssolfa_o2C – associates transiently with TrpA during catalysis to form a functional tryptophan synthase complex. However, in contrast to regular tryptophan synthases, the affinity between the two subunit-types was weak, and activation has been unidirectional from Ssulfo_i2C to TrpA. These results indicate the following ranking for the binding-affinity to TrpA: TrpB2_o < TrpB2_i < TrpB1.

In the course of modelling *trpB *evolution, the relationship between the *trpB *variants has to be made plausible. A possible explanation for the existence of two *trpB *variants would be convergent evolution, *i.e*. the independent development of *trpB1 *and *trpB2 *towards a *trpB *gene. In this case, few residues, which are critical for function, should correspond. However, one would expect these residues embedded into polypeptides, which are relatively dissimilar on the sequence level. In contrast, comparison of TrpB1 and TrpB2 sequences shows that on average 30% of the residues are identical and 40% are similar; compare Table 3. This finding and the conservation of indels makes convergent evolution highly improbable and argues for a common origin of *trpB1 *and *trpB2 *genes.

The most-widely accepted model for the evolution of novel protein functions postulates gene duplication and the generation of a redundant gene copy [41]. It is assumed that evolutionary stress for a copy is largely reduced thus facilitating the evolution of a paralogue with a novel function. This model is based on the notion that negative trade-offs dominate evolutionary processes [42]. According to this model of evolution, one of the *trpB *genes originates from a copy of the ancestral variant. Which of the two existing variants represents the more ancient gene? The arguments listed below suggest that *trpB2 *is the ancestral *trpB *gene.

*i) trpB1 *is not universally distributed among archaea. *Crenarchaeota *possess exclusively *trpB2 *genes. *ii*) A low frequency of amino acids in enzymes required for their synthesis provides selective advantage [40]. In general, *trpB1 *genes contain fewer tryptophan codons than *trpB2 *genes; in *i1_o2 *species, the ratio is 1.6/3.7 *i.e*. less than 0.5. Therefore, *trpB1 *is the more evolved gene. *iii*) The sophisticated inter-subunit communication suggests that the products of *trpB1 *and *trpA1 *of species-types *s1 *or *i1_o2 *are the most efficient enzymes; see [39] and references therein. Hence, TrpB1 is the more optimised and later evolved TrpB variant. *iv*) It has been postulated that ancient enzymes possess broad specificities [43]. The occurrence of *trpB2 *outside *trp *operons argues for either a new function or a broader specificity. In summary, it is plausible to regard *trpB2 *as representing the more ancient variant of *trpB*.

### Modelling the evolution of TrpB

In order to reduce the number of possible alternative scenarios that have to be discussed for modelling the evolution of *trpB*, the following assumptions were made:

*i*) For bacteria, an ancestral *trp *operon of type *trpEGDCFB1A1 *is most likely [10]. Therefore, the existence of a *trpB1 *gene in the bacterial predecessor was taken for granted. In addition, it has been concluded for bacterial *trp *operons that horizontal gene transfer (HGT) did not affect the path of evolutionary history [44].

*ii*) *trpB1*, *trpB2*, *trpA1 *and *trpA2 *have been invented only once. The analysis of multiple sequence alignments (see Figures 5 and 6) shows that the main differences distinguishing the variants are conserved indels. It has been convincingly argued that conserved indels result less likely than *e.g*. point mutations from independent mutational events and provide useful milestones for the identification of evolutionary phases [45]. In addition, the strong coherence seen in the TrpB subtree argues against an independent evolution occurring in parallel for bacteria and archaea. Due to the existence of conserved indels, an evolutionary process *trpB2_i *→ *trpB1 *→ *trpB2_o *or *vice versa *is unlikely too.

*iii*) As has been deduced previously [46], the following order of importance was taken for the processes of genome evolution: gene loss > gene genesis > gene duplication > HGT.

*iv*) The integration of a *trpB *gene into the *trp *operon (or linkage group) was rated less probable than other translocations, gene duplications, gene loss, and mutations. It is presumably very rare that a particular gene gets integrated into a specific gene cluster [47], which is the *trp *operon in the considered case.

*v*) It is unlikely that several recent events of HGT explain the taxonomically widespread occurrence of *trpB2 *genes in bacteria. In bacteria, *trpB2 *genes were found in hyperthermophilic (*Aquificae *and *Thermotogae*) and mesophilic bacteria belonging to the taxonomical groups of *Alpha*- and *Gammaproteobacteria *and *Bacteroides*. The program SIGI [48] identifies genomic islands, *i.e*. gene clusters having a conspicuous codon usage indicating recent HGT events. In none of the considered genomes were *trpB1 *or *trpB2 *genes (both inside and outside operons) elements of such islands.

**Figure 8:**
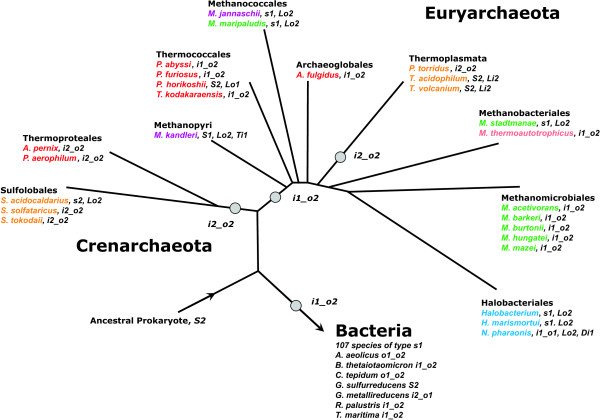
**A parsimonious reconstruction of predecessors**. The phylogenetic tree is based on 16S rRNA sequence comparisons (after Fig. 2 of [49]). For all modern species, their species-type and evolutionary events leading most plausibly from the ancestral predecessor to the current genome content are added. The most probable species-type of predecessors for *Crenarchaeota*, *Euryarchaeota*, and *Bacteria *is given next to the grey circles. Abbreviations for events changing genomic content: Li1 or Lo2, (L)oss of the operon-based *trpB1 *or the non operon-based *trpB2 *gene, respectively. Ti1 (T)ranslocation of the operon based *trpB1*, *Di1 *(D)uplication of the operon-based *trpB1*. The colour of species names indicates the habitat: Hyperthermophiles are given in red, thermoacidophiles in orange, thermophiles in pink, mesophiles in green, halophiles in blue, and species living in a both hyperthermophilic and halophilic environment are given in purple.

In order to model the evolution of tryptophan synthase, a phylogenetic tree based on archeal 16S rRNA sequence comparisons was plotted according to Fig. 2 from [49]. All considered species and their species-types were added. Using the above premises, the most plausible sequence-types of predecessors were determined. These types and evolutionary events needed to infer the modern species-types from the predecessors were added to the tree; see Figure 8.

The most plausible predecessor of all *Crenarchaeota *is of type *i2_o2*; for *Bacteria *and for *Euryarchaeota *it is of type *i1_o2*. Assuming this and excluding *Thermoplasmata *(see below), of the 23 modern archeal species, 14 have the same species-type as their ancestor. Of the 9 species possessing a deviating type, 7 can be explained with a single gene loss, and for only 2 modern species a more complicated genomic rearrangement has to be postulated: Loss of *trpB2 *and dislocation of *trpB1 *has to be postulated for *M. kandleri *(representing *Methanopyri*), which is a *S1 *species. The replacement of *trpB2_o *with a copy of *trpB1 *is necessary to explain the *i1_o1 *genome of *N. pharaonis*. The only euryarcheal class requiring a more complex explanation than gene loss and translocation subsumes *Thermoplasmata*, which possess exclusively *trpB2 *and *trpA2 *genes. The composition of congruency groups (compare Figures 1 and 2) makes a common evolution with *Sulfolobales *or the acquisition of the same *trp *genes probable. The similarity of operon structures supports this assumption: operon structures of *P. torridus *and *Sulfolobales *are identical (compare Panel B of Figure 7). For *T. acidophilum*, a large amount of HGT with *S. solfataricus*, which is found in the same habitat, has been made plausible [50]. In summary, a common evolutionary history of *trpB2 *and *trpA2 *genes of *Sulfolobales *and *Thermoplasmata *is highly plausible, proposing for both taxonomical classes an ancestor of species-type *i2_o2*. Assuming an *i2_o2 *ancestor, gene loss is sufficient to explain the genome composition of all modern *Thermoplasmata*.

**Figure 9:**
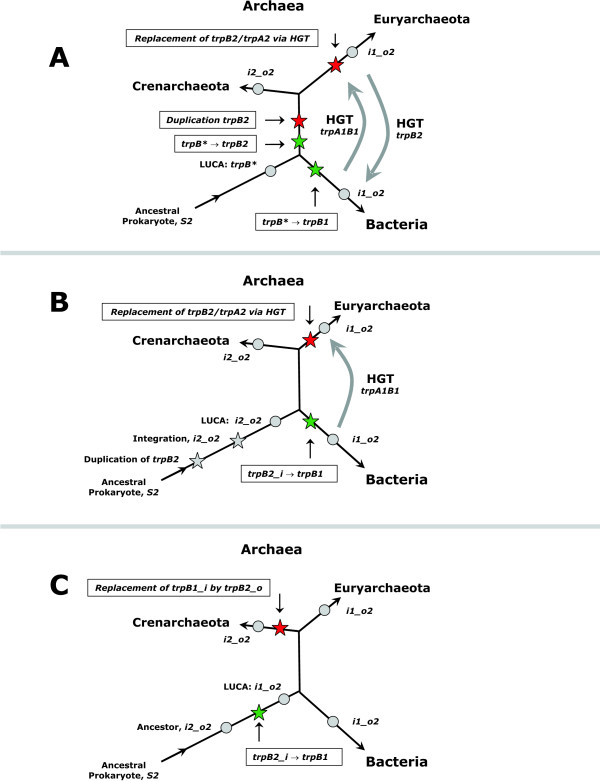
**Alternative models of *trpB *evolution**. Model A assumes that a single and intermediate trpB* gene existed in the last universal common ancestor (LUCA) of bacteria and archaea. The evolution of the *trpB2 *gene is considered an archeal and that of the *trpB1 *gene is considered a bacterial invention. The occurrence of *trpA1 *and *trpB1 *genes in archaea and of *trpB2 *genes in bacteria are explained by a twofold horizontal gene transfer (HGT). A duplication of *trpB2 *in an ancient archeal genome has been postulated to explain the existence of the non operon-based *trpB2*. Models B and C propose two alternatives for the evolution of the LUCA. Model B assumes that the evolution *trpB2 *→ *trpB1 *occurred in an early bacterial species after the divergence of bacteria and archaea. The replacement of linkage group *trpB2A2 *by *trpB1A1 via *HGT was postulated to account for the euryarcheal predecessor of type *i1_o2*. Model C assumes that the evolution *trpB2 *→ *trpB1 *occurred before the divergence of bacteria and archaea. Hence, the replacement of an operon-based *trpB1 *by a *trpB2 *gene and the evolution *trpA1 *→ *trpA2 *was postulated for the crenarcheal ancestor. For acronyms of species-types, see legend of Table 2. Distances are arbitrary and do not represent evolutionary time intervals. Stars indicate events of genomic rearrangements, circles filled in grey represent ancient predecessors.

Based on these predecessors, three alternatives explaining the evolution and distribution of *trpB *species starting from the last universal common ancestor (LUCA) of bacteria and archaea were deduced (Figure 9, Panels A – C). In the following paragraph, the plausibility of these alternatives will be discussed. The rest of this paragraph is used to elucidate the three alternatives.

In Panel A of Figure 9, the existence of an ancestral trpB*, an intermediate of *trpB1 *and *trpB2 *was postulated for the LUCA. trpB* might then have diverged into a bacterial *trpB1 *and an archeal *trpB2 *variant. To explain the existence of a non operon-based *trpB2 *in archaea, a duplication of *trpB2 *is necessary. The advent of an euryarcheal *i1_o2 *predecessor requires the replacement of linkage group *trpB2A2 *with *trpB1A1 via *HGT from bacteria to archaea. The occurrence of *trpB2 *in bacterial genomes demands an early transfer of *trpB2 *from an archeal to a bacterial predecessor.

Panel B of Figure 9 depicts an alternative model for the evolution of the LUCA towards the bacterial and archeal ancestors. As introduced above, gene duplication is regarded the first step for evolving a novel gene function. In addition, *trpB2 *must be considered to represent the more ancient variant of *trpB*. Therefore, the evolution towards the LUCA of bacteria and archaea is most plausibly explained by the duplication of a non operon-based *trpB2 *gene, which was subsequently integrated into the *trp *operon and constituted an ancient linkage group *trpB2A2*. This makes a common ancestor of type *i2_o2 *plausible. These considerations are the basis for further reconstructing the evolution of predecessors. In Panels B and C, two alternatives are given.

In Panel B, it is assumed that the LUCA was of type *i2_o2 *and that the evolution *trpB2 *→ *trpB1 *occurred in an early bacterial species. In this case, species-types of the LUCA and the crenarcheal predecessor are identical. To explain the advent of an euryarcheal predecessor of type *i1_o2*, an ancient event of HGT from *Bacteria *to *Archaea *has to be postulated for the acquisition of the linkage group *trpB1A1*, which replaced *trpB2A2*.

In Panel C, it is assumed that the LUCA was of type *i1_o2*, *i.e*. the evolution *trpB2 *→ *trpB1 *occurred earlier than the speciation of *Bacteria *and *Archaea*. In this case, the species-types of the LUCA and the predecessors of *Bacteria *and *Euryarchaeota *are identical. However, a replacement of *trpB2_i *by *trpB2_o *is necessary to constitute the crenarcheal predecessor.

### How plausible are these three models?

Model A requires at least two ancient events of HGT to explain the occurrence of *trpB2 *in *Bacteria *and of *trpA1B1 *in *Euryarchaeota*. The phenomenon of non-orthologous displacement *in situ *is well-characterised [51,52]. In addition to HGT, a duplication of the *trpB2 *gene is needed for the predecessor of *Archaea*. This model is not the most parsimonious one: Model B demands only one HGT event, the ancient acquisition of the linkage group *trpB1A1 *by an euryarcheal predecessor.

Model C postulates a LUCA of species-type *i1_o2*. The sophisticated inter-subunit communication clearly suggests that products of *trpB1 *and *trpA1 *genes are the most specialised and most recently evolved tryptophan synthases; see [39] and references therein. Thus, the replacement of *trpB1 *with *trpB2*, which is needed to explain the existence of a crenarcheal predecessor of type *i2_o2*, would – with respect to protein-protein interaction – lead to a less optimal tryptophan synthase. This seems unlikely, if one presumes the sustained need for tryptophan synthesis in *Crenarchaeota*.

**Figure 10:**
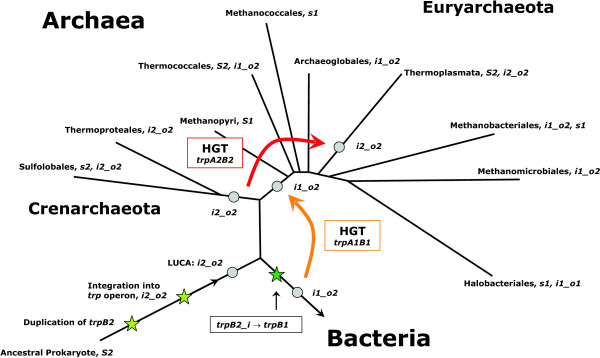
**Composite model of *trpB *evolution**. Upon duplication and integration of an ancient *trpB2 *gene into the *trp *operon, the last universal common ancestor (LUCA) of bacteria and archaea was of species-type *i2_o2*. In a bacterial ancestor, the evolution of a linkage group *trpB1A1 *occurred. *Via *horizontal gene transfer (HGT), an euryarcheal ancestor acquired this linkage group, which gave rise to a predecessor of type *i1_o2*. *Thermoplasmata *acquired *trpA2 *and *trpB2 *genes in an ancient event of HGT. For all taxonomical orders, species-types of current species are given. *S2 *species possess exactly one, non operon-based *trpB2 *gene, *s2*: ditto, the gene is located inside the *trp *operon. *trpB1 *was treated analogously. *i2_o2 *are species possessing a *trpB2 *gene inside and a second *trpB2 *outside the operon, *i1_o2 *are species with an operon-based *trpB1 *and a non operon-based *trpB2*, and *i1_o1 *are species possessing an operon-based and at least one non operon-based *trpB1*.

In contrast, model B postulates the replacement of a (less evolved) *trpB2_i *by a *trpB1 *for the euryarcheal predecessor and does not require the replacement of a *trpB1 *by a *trpB2 *for the crenarcheal predecessor. The case of *Thermoplasmata *makes clear that in a thermophilic or hyperthermophilic environment *trpB2 *and *trpA2 *genes are favoured over *trpB1 *and *trpA1*. There is evidence that the LUCA was a thermophilic or hyperthermophilic species [34,53,54]. Therefore, it is more probable to expect a LUCA of species-type *i2_o2*. In summary, considering parsimony arguments and the assumption that negative trade-offs dominate evolutionary processes [42], model B is the likelier one. Figure 10 summarises the most parsimonious scenario explaining the composition of modern archeal *trp *operons: Assuming that the LUCA was of type *i2_o2*, and that *trpB1 *was a bacterial invention, besides gene loss, which is a frequent evolutionary event, two cases of ancient HGT are sufficient to explain the distribution of *trpA *and *trpB *species in current archeal genomes.

## Conclusion

In archeal genomes, various stages of *trpB *function have been conserved. Most plausibly, *trpB2 *represents the ancestral variant of *trpB *genes. With respect to TrpA/TrpB communication and cooperativity, the situation observed in *S2 *species (*T. acidophilum *and *T. volcanium*) is probably the least complex one. Similarly archaic are the non operon-based *trpB2 *genes of *Sulfolobaceae*, whereas the operon-based *trpB *genes are more evolved. *s1 *and *i1_o2 *species possess highly cooperative synthases. Thus, the archeal tryptophan synthase (especially *trpB *variants) constitutes a model system for the study of protein complex formation. Due to different environmental conditions, several stages of cooperativity have been conserved, which allow to characterise the progress of *trpA *– *trpB *coevolution based on gene expression and on functional cooperativity.

## Materials

### Genomes and protein sequences

Genomic content was determined by analysing version 6.2 of the STRING database [30].

All protein sequences were downloaded *via *the "Genome Project" database of the NCBI [55], which allows to access completely sequenced genomes. Respective COG tables were consulted to determine the COG group of genes [29] and to download sequences. Genes originating from the following completely sequenced genomes were analysed (abbreviations used for Figures and accession numbers of genomes in brackets):

### Crenarchaeota

*Aeropyrum pernix *K1 (Aperni, NC_00854), *Pyrobaculum aerophilum *str. IM2 (Paerop, NC_003364), *Sulfolobus acidocaldarius *DSM 639 (Sacido, NC_007181), *Sulfolobus solfataricus *P2 (Ssolfa, NC_002754), *Sulfolobus tokodaii *str. 7 (Stokod, NC_003106).

### Euryarchaeota

*Archaeoglobus fulgidus *DSM 4304 (Afulgi, NC_000917), *Haloarcula marismortui *ATCC 43049 (Hmaris, NC_006396), *Halobacterium *sp. NRC-1 (Halob, NC_002607), *Methanocaldococcus jannaschii *DSM 2661 (Mjanna, NC_000909), *Methanococcoides burtonii *DSM 6242 (Mburto, NC_007955), *Methanococcus maripaludis *S2 (Mmarip, NC_005791), *Methanopyrus kandleri *AV19 (Mkandl, NC_003551), *Methanosarcina acetivorans *C2A (Maceti, NC_003552), *Methanosarcina barkeri *str. Fusaro (Mbarke, NC_007355), *Methanosarcina mazei *Go1 (Mmazei, NC_003901), *Methanosphera stadtmanae *DSM 3091 (Mstadt, NC_007681), *Methanospirillum hungatei *JF-1 (Mhunga, NC_007796) *Methanothermobacter thermautotrophicus *str. Delta H. (Mtherm, NC_000916), *Natronomonas pharaonis *DSM 2160 (Nphara, NC_007426), *Picrophilus torridus *DSM9790 (Ptorri, NC_005877), *Pyrococcus abyssi *GE5 (Pabyss, NC_000868), *Pyrococcus furiosus *DSM 3638 (Pfurio, NC_003413), *Pyrococcus horikoshii *OT3 (Phorik, NC_000961), *Thermococcus kodakaraensis *KOD1 (Tkodak, NC_006624), *Thermoplasma acidophilum *DSM1728 (Tacido, NC_002578), *Thermoplasma volcanium *GSS1 (Tvolca, NC_002689).

### Bacteria

*Escherichia coli *K-12 (Ecoli, NC_000913), *Geobacter metallireducens *GS-15 (Gmetal, NC_007517), *Geobacter sulfurreducens *PCA (Gsulfu, NC_002939), *Thermotoga maritima *(Tmarit, NC_00853).

## Methods

### Generating multiple sequence alignments

For the generation of multiple sequence alignments (MSAs) the program M-Coffee [32] was used. It combines the output of nine individual MSA methods for the generation of a "meta"-MSA. M-Coffee has been shown to outperform all individual methods of MSA generation [32].

### Annotating multiple sequence alignments

For each position in a MSA, residue conservation, secondary structure, the location of the interface area, active sites and residues, which are characteristic for sequence types, were determined and plotted. 3D-data were deduced from the PDB-file 1WDW, describing the TrpA/TrpB complex of *P. furiosus *[18]. For 2D-structure prediction, Jpred [37] was used. SDPpred [38] was utilised to identify those residues, which distinguished sequence groups due to their skewed or bimodal distribution. Annotations referring active site residues were deduced from the PDBsum page [56,57], interface residues were annotated according to the *Protein interfaces, surfaces and assemblies service PISA *[58,59]. Both services were located at the webserver of the European Bioinformatics Institute (EMBL-EBI).

### Creating and evaluating phylogenetic trees

SplitsTrees4 [33], a frame-work for phylogenetic analyses, was used to generate and analyse phylogenetic trees. MSAs originating from M-Coffee were utilised to calculate maximum likelihood protein distance estimates based on a JTT [60] model. The bio-neighbour joining approach [61] was used to generate trees. Resulting trees were analysed by bootstrapping (1000 replications each).
